# Chronic Arsenic Toxicity Without Classical Cutaneous Manifestations: A Case Report

**DOI:** 10.7759/cureus.113344

**Published:** 2026-07-25

**Authors:** Sunil K Jyani, Ravi Godara, Shrikant Choudhary, Raman Sharma

**Affiliations:** 1 Geriatric Medicine, Mahatma Gandhi University of Medical Sciences and Technology, Jaipur, IND; 2 Internal Medicine, Mahatma Gandhi University of Medical Sciences and Technology, Jaipur, IND

**Keywords:** arsenicosis, bhasma, chronic arsenic toxicity, cutaneous manifestation, herbal medicine, peripheral neuropathy

## Abstract

Chronic arsenic toxicity can present with gastrointestinal and neurological symptoms, and diagnosis is often difficult without characteristic cutaneous features. This report describes a 55-year-old woman with a three-month history of progressively worsening small bowel-type diarrhea that progressed to frequent episodes of melena. She developed painful stocking-distribution peripheral neuropathy with slipper slippage, followed by progressive lower-limb weakness that ultimately resulted in loss of ambulation. She had a seven-year history of unsupervised use of herbal preparations and Ayurvedic Bhasma. Despite comprehensive evaluation, the diagnosis remained unclear because classical cutaneous signs of chronic arsenicosis were absent. A thorough medication history led to heavy metal screening (urinary arsenic level), which confirmed arsenic toxicity. Discontinuation of the suspected herbal preparations and supportive care, without chelation therapy, resulted in complete resolution of gastrointestinal symptoms and anemia, gradual neurological recovery, and restoration of independent ambulation. This case highlights the need to consider chronic arsenic toxicity in patients with unexplained chronic diarrhea and painful peripheral neuropathy, even when characteristic skin findings are absent. A detailed inquiry into the use of complementary and alternative medicine is crucial, as early identification and removal of the source of exposure can facilitate significant clinical recovery.

## Introduction

Traditional herbal and Bhasma preparations are recognized as potential iatrogenic sources of arsenic exposure, which may result in both acute and chronic toxicity [1,2]. Acute arsenic poisoning typically presents with severe gastrointestinal symptoms, including vomiting, abdominal pain, and profuse diarrhea [3]. In contrast, chronic arsenic toxicity is a multisystem disorder that classically manifests with cutaneous findings such as raindrop pigmentation, palmoplantar hyperkeratosis, and Mee’s lines, as well as neurological, hepatic, gastrointestinal, respiratory, renal, and neoplastic complications [4]. We report a case of chronic arsenic exposure, presumed to be related to herbal preparation ingestion, presenting with enterocolitis, anemia, and painful peripheral neuropathy, despite the absence of classical dermatological features of arsenicosis. This case is notable for its atypical presentation without the usual cutaneous signs of chronic arsenicosis.

## Case presentation

A 55-year-old woman, a homemaker with a past medical history of hypothyroidism for 18 years, presented with a three-month history of progressively worsening gastrointestinal symptoms. She initially presented with large-volume, loose, and watery stools occurring five times per day after eating. These episodes were accompanied by cramping abdominal pain around the umbilical area. Over the preceding 15 days, she noticed that the frequency increased to more than 10 times per day, and that the stools became dark and foul-smelling. Over the same three-month period, she developed gradually worsening burning pain in both lower limbs, beginning in the toes and later extending up to the mid-thigh level. It was associated with difficulty walking, including slipping out of her slippers without realizing it. She also developed progressive weakness in both lower limbs, first noticed as toe dragging, followed by difficulty squatting and rising from a chair. She reported no history suggestive of upper limb, cranial nerve, bladder involvement, autonomic symptoms, muscle fasciculations, loss of consciousness, or seizures. 

She denied having fever, hematemesis, rashes, jaundice, oral ulcers, joint pain, hematuria, night sweats, or weight loss. She had no diabetes, hypertension, chronic liver disease, or chronic kidney disease, and her thyroid function remained stable on replacement therapy. She was a nonsmoker and nonalcoholic and followed a mixed diet. There was no history of similar complaints among her family members or neighbors, and she lived in an urban pucca house with access to safe, reverse-osmosis drinking water. She was also using unspecified herbal preparations and bhasma that had been prescribed by a qualified practitioner seven years earlier for hypothyroid and urinary symptoms; however, she had continued taking these preparations without expert supervision.

On presentation to the emergency department, she was alert and hemodynamically stable. Pallor was present, and no cutaneous or mucosal signs, including raindrop pigmentation, palmar-plantar hyperkeratosis, Mees’ lines on the nails, or gingival hyperpigmentation, were seen. Diffuse tenderness was present on deep palpation of the abdomen, with no guarding, rigidity, or organomegaly. Higher mental functions and cranial nerves were normal. Motor testing showed bilateral lower-limb weakness, with power graded 4/5 at the hips, 3/5 at the knees, and 2/5 at the ankles for dorsiflexion and plantarflexion. Deep tendon reflexes were 2+ in both upper limbs, 1+ at the knees, and absent at the ankles. Temperature and touch sensations were reduced by approximately 30% at the feet, 50% at the shins, and 70% at mid-thigh level, with preservation above mid-thigh. Joint position sense was lost up to the ankle joints. The rest of the neurological and organ system examinations were unremarkable.

Initial investigations showed normocytic, normochromic anemia with a hemoglobin of 7.4 g/dL; corrected reticulocyte count, serum ferritin, and transferrin saturation were 1.72%, 272 ng/mL and 18.5%, respectively. The leukocyte count was elevated (15,790/µL) with neutrophilia and toxic granulations, without atypical cells or blasts. Serum electrolytes, renal function, liver function, thyroid function tests, lactate dehydrogenase (LDH), and creatine phosphokinase (CPK) were within normal limits. Hepatitis B surface antigen (HBsAg), human immunodeficiency virus (HIV) serology, and anti-hepatitis C virus (anti-HCV) antibodies were nonreactive. Vitamin B12, alpha-fetoprotein (AFP), cancer antigen 19-9 (CA 19-9), and carcinoembryonic antigen (CEA) were normal. Autoimmune testing, including antinuclear antibody (ANA), antineutrophil cytoplasmic antibody (ANCA), direct Coombs test (DCT), and immunoglobulin A anti-tissue transglutaminase (IgA anti-tTG) antibodies, were negative. Serum and urine protein electrophoresis (SPEP/UPEP) showed no monoclonal band detected. Stool occult blood was positive, and inflammatory markers were elevated (CRP 70 mg/L, ESR 60 mm/hr; Table 1)

**Table 1:** Baseline and follow-up laboratory investigations with reference ranges AST, aspartate aminotransferase; ALT, alanine aminotransferase; ALP, alkaline phosphatase; TSH, thyroid-stimulating hormone; LDH, lactate dehydrogenase; CK/CPK, creatine kinase/creatine phosphokinase; HbA1c, glycated hemoglobin; CRP, C-reactive protein; HBsAg, hepatitis B surface antigen; HIV, human immunodeficiency virus; HCV, hepatitis C virus; ICP-MS, inductively coupled plasma mass spectrometry.

Laboratory parameter	Patient value	Reference range
Hemoglobin	7.4 g/dL	12.0–15 g/dL
Total leukocyte count	15,790/µL	4,000–10,000/µL
Neutrophils	82.2%	40–80%
Serum sodium	133.4 mmol/L	135–145 mmol/L
Serum potassium	3.6 mmol/L	3.5–5.0 mmol/L
Serum urea	18 mg/dL	Adult: 15-45 mg/dL
Serum creatinine	0.7 mg/dL	0.6–1.1 mg/dL
Serum uric acid	2.3 mg/dL	2.5-6.2 mg/dL
Total bilirubin	0.6 mg/dL	0.3–1.2 mg/dL
AST	44.9 U/L	10–40 U/L
ALT	20.7 U/L	7–56 U/L
ALP	109.2 U/L	44–147 U/L
Free T3	2.47 pg/ml	2.77-5.27 pg/mL
Free T4	2.08 ng/dL	0.8–1.8 ng/dL
TSH	3.2 mIU/L	0.4–4.0 mIU/L
Lactate dehydrogenase (LDH)	202.3 U/L	140–280 U/L
Creatine phosphokinase (CK/CPK)	50.9 U/L	30–170 U/L
Vitamin B12	>1000 pg/ml	200–900 pg/mL
Ferritin	272 ng/ml	50-150 ng/ml
Transferrin saturation	18.5%	20-50%
Corrected reticulocyte count	1.72%	0.5-2.5%
HbA1C	<4.0 %	4.5-6.30%
C-reactive protein (CRP)	70 mg/L	Upto 10 mg/L
Erythrocyte sedimentation rate (ESR)	60 mm/hour	Female: <30 mm/hour
Stool occult blood	Positive	Negative
HBsAg	Negative	Negative
HIV serology	Negative	Negative
Anti-HCV antibody	Negative	Negative
Antinuclear antibody (ANA)	Negative (1:80)	Negative
Antineutrophil cytoplasmic antibody (ANCA)	Negative	Negative
Direct Coombs test (DCT)	Negative	Negative
IgA anti-tissue transglutaminase (IgA anti-tTG)	4.2 U/mL	<20.0 U/mL
Serum protein electrophoresis (SPEP)	No M band	No monoclonal band
Urine protein electrophoresis (UPEP)	No M band	No monoclonal band
Serum cortisol	19.5 µg/dL	5-25 µg/dL
Alpha-fetoprotein (AFP)	1.73 IU/mL	<10 IU/mL
Carbohydrate antigen 19-9 (CA 19-9)	40.0 U/mL	<37 U/mL
Carcinoembryonic antigen (CEA)	4.44	<5 ng/mL
Urinary arsenic (ICP-MS)	184.15 µg/L	<35 µg/L
Hemoglobin (2 weeks after cessation)	10.2 g/dL	12.0–15 g/dL
Urinary arsenic (ICP-MS) (12 weeks after cessation)	Not detected	<35 µg/L

**Figure 1:**
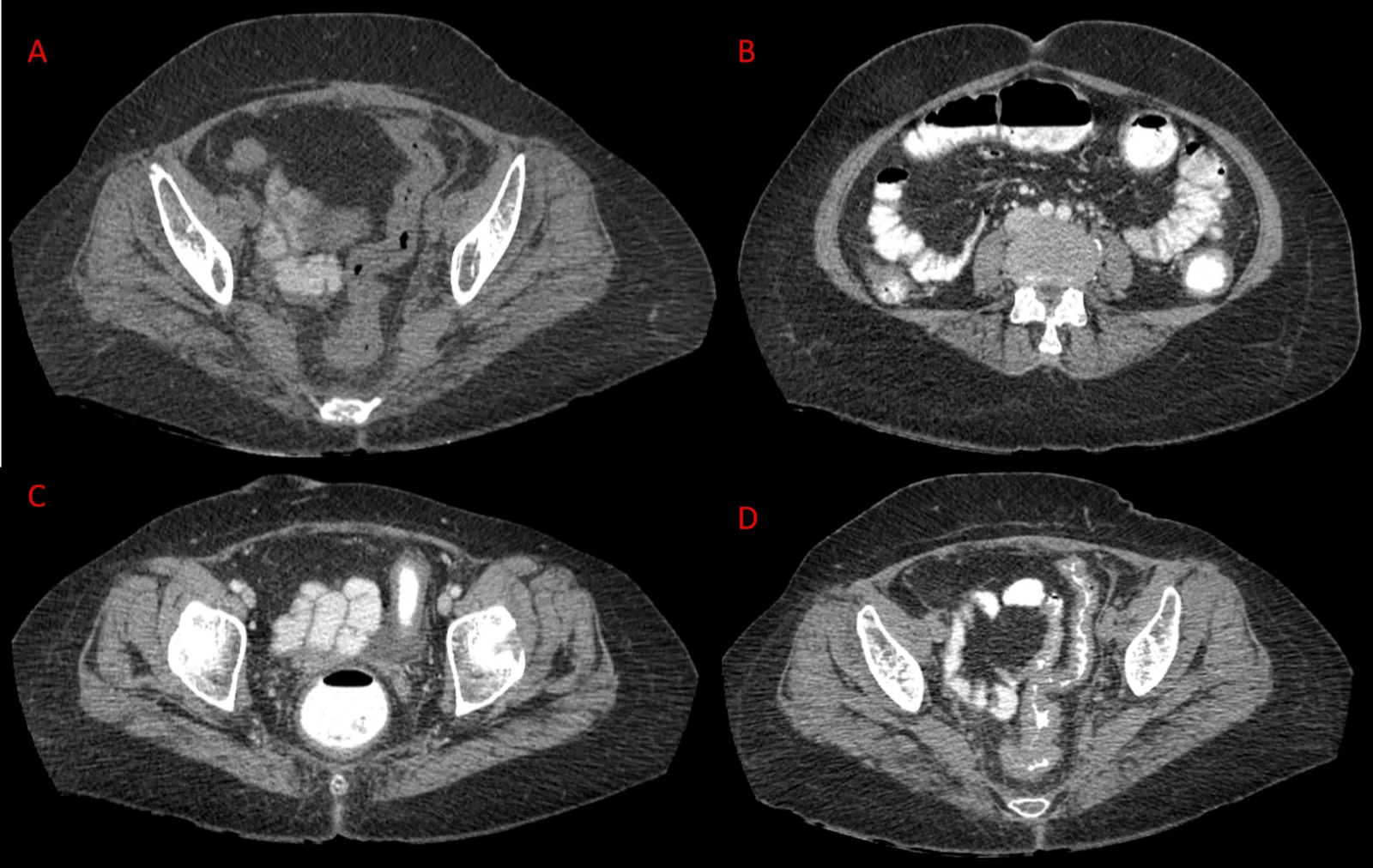
Contrast-enhanced computed tomography (CT) of the abdomen and pelvis (A-D) Diffuse mild circumferential wall thickening of the rectum, sigmoid colon, descending colon, and distal transverse colon, with mild pericolonic fat stranding.

Upper gastrointestinal endoscopy was normal. Contrast-enhanced CT of the abdomen showed diffuse, mild, circumferential wall thickening of the rectum, sigmoid colon, descending colon, and distal transverse colon, with mild pericolonic fat stranding, suggestive of nonspecific colitis (Figure 1).

In view of these radiological findings and the initial clinical presentation, an infective etiology was suspected; antibiotics piperacillin-tazobactam and metronidazole were started. Stool color normalized and leukocytosis resolved within five days of therapy, but diarrhea and neurological deficits persisted. Further evaluation was undertaken and nerve conduction studies showed bilateral symmetrical sensorimotor axonal polyneuropathy, with markedly reduced compound muscle action potential (CMAP) and sensory nerve action potential (SNAP) amplitudes, and relatively preserved conduction velocities. Subcutaneous fat-pad biopsy revealed no amyloid deposition, but the patient declined cerebrospinal fluid examination and colonoscopy.

**Figure 2:**
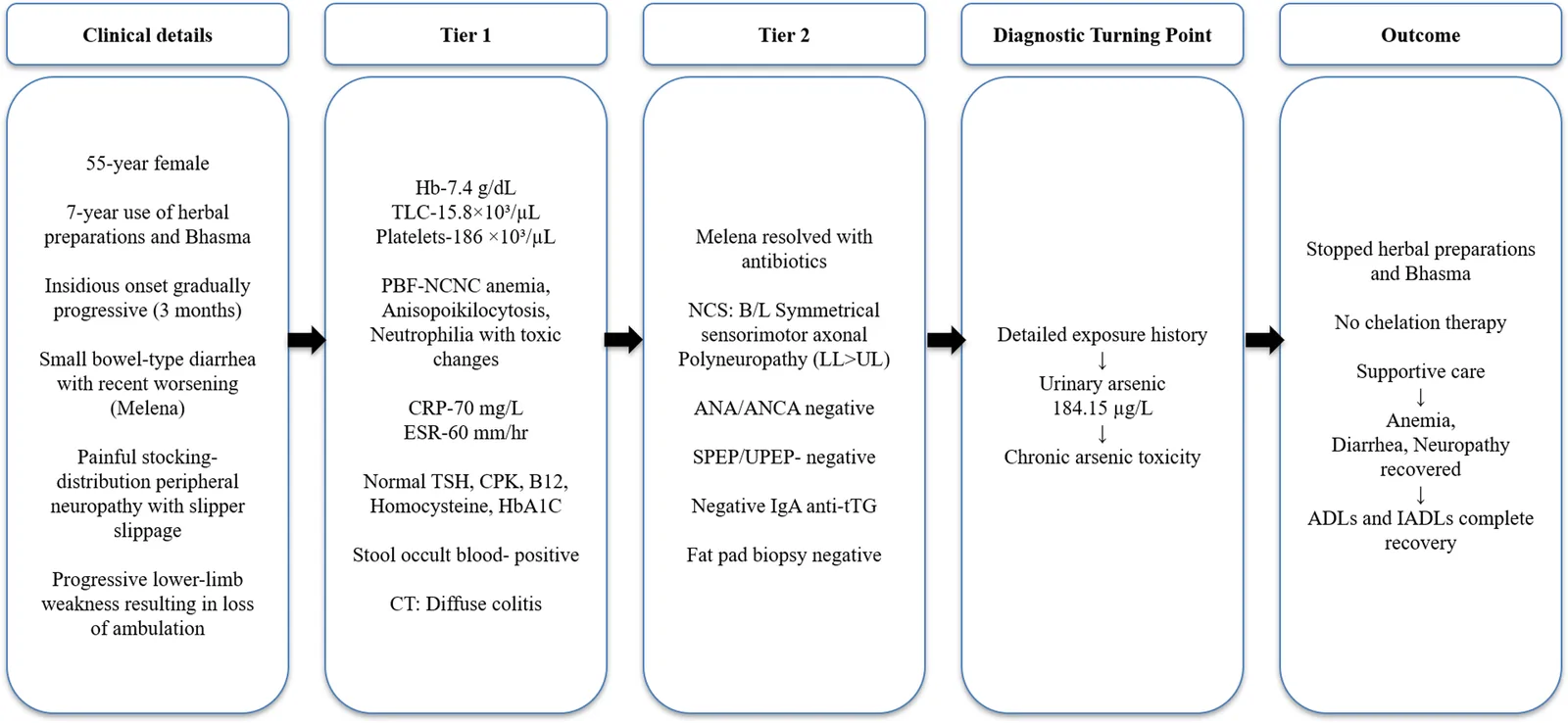
Clinical algorithm highlighting the diagnostic journey of the patient with chronic arsenic toxicity Hb, hemoglobin; TLC, total leukocyte count; PBF, peripheral blood film; NCNC,normocytic normochromic; CRP, C-reactive protein; ESR, erythrocyte sedimentation rate; TSH, thyroid-stimulating hormone; CK, creatine kinase; B12, vitamin B12; HbA1c, glycated haemoglobin; CT, computed tomography; NCS, nerve conduction study; B/L, bilateral; LL, lower limbs; UL, upper limbs; ANA, antinuclear antibody; ANCA, antineutrophil cytoplasmic antibody; SPEP, serum protein electrophoresis; UPEP, urine protein electrophoresis; IgA anti-tTG, immunoglobulin A anti-tissue transglutaminase antibody; ADLs, activities of daily living; IADLs, instrumental activities of daily living. Image credit: This figure is original work created by the authors for this manuscript and was created using Microsoft PowerPoint (Microsoft Corp., Redmond, WA, USA).

With no unifying diagnosis and a history of alternative medicine use, heavy-metal screening by inductively coupled plasma mass spectrometry (ICP-MS) was performed. It revealed a critically elevated urinary arsenic level of 184.15 mcg/L (reference <35 mcg/L). A final diagnosis of chronic arsenic toxicity presenting with bilateral symmetrical sensorimotor axonal polyneuropathy, anemia, and arsenic-associated enterocolitis, possibly exacerbated by secondary infection, was considered. It led to a detailed re-examination of her exposure history; family members and neighbors showed no similar symptoms, and no environmental sources of arsenic exposure were identified. She had taken Bhasma with milk irregularly for seven years, using approximately a 5 gm pouch over four to five months, with the last dose taken on the sixth day of admission to our center. All herbal medications were stopped, and considering this chronic exposure, no chelating agents were used, and she was closely monitored. Over the following weeks, her gastrointestinal symptoms resolved, and her hemoglobin increased to 10.2 g/dL within two weeks. Urinary arsenic became undetectable 12 weeks after cessation of exposure. By 30 weeks, she was able to walk independently. At the time of drafting this report, 56 weeks after diagnosis, she remains independent in all activities of daily living (ADL) and instrumental activities of daily living (IADL) and continues only thyroxine 125 µg daily (Figure 2).

## Discussion

Arsenic is a recognized contaminant or ingredient in certain traditional medicinal preparations across various countries, including herbal and Bhasma formulations [3-5]. Chronic arsenic toxicity is typically associated with cutaneous manifestations such as melanosis, palmoplantar keratosis, and, in advanced cases, Bowen’s disease or cutaneous malignancy [6]. However, the absence of these dermatological signs does not preclude the diagnosis. In the present case, the principal manifestations included gastrointestinal illness, anemia, and bilateral symmetrical sensorimotor axonal polyneuropathy, corroborated by a markedly elevated urinary arsenic level (Table 2).

**Table 2:** Clinical manifestations of acute and chronic arsenic toxicity compared with the present case [3,4,7]

Clinical feature	Acute arsenic toxicity	Chronic arsenic toxicity	Present case
Onset	Hours to days after exposure	Months to years of exposure	Seven-year history of exposure, with progressive symptoms over 3 months
Gastrointestinal manifestations	Severe nausea, vomiting, profuse watery ("rice-water") diarrhea, abdominal pain	Chronic diarrhea, abdominal discomfort, weight loss	Progressive small-bowel type diarrhea with progression to melena and abdominal pain
Neurological manifestations	Acute encephalopathy (severe cases)	Symmetrical sensorimotor axonal peripheral neuropathy	Painful bilateral symmetrical sensorimotor axonal polyneuropathy with lower-limb weakness
Cutaneous manifestations	Absent	Raindrop pigmentation, palmar-plantar hyperkeratosis, Mees' lines	Absent
Cardiovascular manifestations	Hypotension, QT prolongation, arrhythmia	Peripheral vascular disease	Absent
Hematological manifestations	Hemolysis, leukopenia	Anemia (normocytic normochromic), bone marrow suppression	Severe anemia (normocytic normochromic), no evidence of hemolysis
Hepatic/Renal involvement	Acute hepatic or renal injury	Chronic liver dysfunction, non-cirrhotic portal fibrosis	Absent
Exposure source	Accidental ingestion, contaminated food/water, poisoning	Contaminated groundwater, occupational exposure, traditional/herbal medicines	Long-term consumption of herbal preparations and Bhasma
Diagnostic clue	Acute toxic exposure history with rapid onset of symptoms	Characteristic skin manifestations often prompt evaluation	Diagnosis established after detailed medication history and elevated urinary arsenic despite absence of classical skin findings

The multisystem involvement observed is attributable to arsenic’s cellular toxicity, which occurs through binding to sulfhydryl groups, inhibition of pyruvate dehydrogenase, impaired mitochondrial oxidative phosphorylation, reduced ATP production, and induction of oxidative stress. These pathophysiological mechanisms may result in distal axonal neuropathy, gastrointestinal mucosal injury, and reversible bone marrow suppression [8-10].

While the CT findings initially suggested inflammatory bowel disease (IBD) as a differential diagnosis, the spontaneous resolution of gastrointestinal symptoms after withdrawal of the herbal medications, in the absence of disease-specific treatment, argued strongly against the IBD. Diagnosis of chronic arsenic toxicity primarily depends on detecting elevated urinary arsenic concentrations, with arsenic speciation preferred to differentiate toxic inorganic arsenic from organic arsenic associated with seafood intake. Hair, nail, environmental, or product testing can help confirm exposure when urinary arsenic levels have normalized [11].

Management focuses on early recognition and complete elimination of the source of exposure, accompanied by supportive care tailored to the affected organ systems. Mazumdar et al. demonstrated clinical benefit of 2,3-dimercapto-1-propanesulfonate (DMPS) in patients with arsenicosis and ongoing exposure [12]. However, the same author reported in a separate randomized controlled trial that 2,3-dimercaptosuccinic acid (DMSA) was not effective [13]. Overall, evidence for chelation in chronic arsenic toxicity remains limited and inconsistent; however, it may be considered in selected patients with recent exposure, severe symptoms, or progressive organ dysfunction [10]. Chelation is most effective when therapy is initiated early, before extensive tissue redistribution, and decreases with delayed treatment. Urinary arsenic elevation supports recent exposure but should not alone determine chelation, as decisions must also account for clinical severity, exposure status, and treatment risks, including increased urinary loss of essential trace elements such as zinc and copper. In this patient, the urinary arsenic concentration was 184.15 μg/L. However, the exposure source had already been discontinued on the sixth day of admission (14 days prior to diagnosis), toxicity was not progressing, and the patient showed spontaneous gastrointestinal, hematological, and neurological improvement with supportive care alone. Laboratory confirmation was obtained approximately two weeks after sample collection, and chelating agents became available only about one month after diagnosis, further limiting the potential benefit of therapy. Conservative management without chelation was therefore considered appropriate and resulted in gradual recovery.

This report has several important limitations. These include the lack of analysis of the consumed preparations, which prevented definitive confirmation of the exact source and chemical form of arsenic; the absence of urinary arsenic speciation, although recent seafood intake was excluded by dietary history; and the patient’s refusal of colonoscopy and cerebrospinal fluid examination, which limited comprehensive evaluation of gastrointestinal and neurological differentials.

## Conclusions

Chronic heavy metal toxicity should be considered in patients presenting with unexplained multisystem involvement, even in the absence of classical cutaneous signs. A history of prolonged use of unregulated traditional or alternative medicines should prompt evaluation for toxic metal exposure. Early identification and complete elimination of the source of arsenic remain the cornerstones of treatment. Chelation therapy is not routinely indicated in chronic arsenic exposure and should be reserved for selected cases. This case report highlights the need for stricter regulation, quality control, and post-marketing surveillance of traditional medicinal products, with safety standards equivalent to those applied to modern pharmaceutical therapies.
